# A Simple Composite Phenotype Scoring System for Evaluating Mouse Models of Cerebellar Ataxia

**DOI:** 10.3791/1787

**Published:** 2010-05-21

**Authors:** Stephan J. Guyenet, Stephanie A. Furrer, Vincent M. Damian, Travis D. Baughan, Albert R. La Spada, Gwenn A. Garden

**Affiliations:** Department of Biochemistry, University of Washington; Department of Neurology, University of Washington; Division of Genetics, Departments of Pediatrics and Cellular and Molecular Medicine, and the Institute for Genomic Medicine, University of California, San Diego - Rady Children’s Hospital

## Abstract

We describe a protocol for the rapid and sensitive quantification of disease severity in mouse models of cerebella ataxia. It is derived from previously published phenotype assessments in several disease models, including spinocerebellar ataxias, Huntington s disease and spinobulbar muscular atrophy. Measures include hind limb clasping, ledge test, gait and kyphosis. Each measure is recorded on a scale of 0-3, with a combined total of 0-12 for all four measures. The results effectively discriminate between affected and non-affected individuals, while also quantifying the temporal progression of neurodegenerative disease phenotypes. Measures may be analyzed individually or combined into a composite phenotype score for greater statistical power. The ideal combination of the four described measures will depend upon the disorder in question. We present an example of the protocol used to assess disease severity in a transgenic mouse model of spinocerebellar ataxia type 7 (SCA7).

Albert R. La Spada and Gwenn A. Garden contributed to this manuscript equally.

**Figure:**
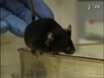


## Protocol

To prevent bias, the experimenter performing the assessments should not have knowledge of the animal's genotype. Individual measures are scored on a scale of 0-3, with 0 representing an absence of the relevant phenotype and 3 representing the most severe manifestation. Each test is performed multiple times to ensure that the score is reproducible. Obesity will complicate the interpretation of all measures described. The investigator may wish to weigh mice following phenotype scoring to assess the possible role of adiposity in the results. 

### Ledge test

The ledge test is a direct measure of coordination, which is impaired in cerebellar ataxias and many other neurodegenerative disorders. This measure is the most directly comparable to human signs of cerebellar ataxia.

1. Lift the mouse from the cage and place it on the cage's ledge. Mice will typically walk along the ledge and attempt to descend back into the cage.
2. Observe the mouse as it walks along the cage ledge and lowers itself into its cage. A wild-type mouse will typically walk along the ledge without losing its balance, and will lower itself back into the cage gracefully, using its paws. This is assigned a score of 0. If the mouse loses its footing while walking along the ledge, but otherwise appears coordinated, it receives a score of 1. If it does not effectively use its hind legs, or lands on its head rather than its paws when descending into the cage, it receives a score of 2. If it falls off the ledge, or nearly so, while walking or attempting to lower itself, or shakes and refuses to move at all despite encouragement, it receives a score of 3. Some mice will require a gentle nudge to encourage them to walk along the ledge or descend into the cage.
3. Record the ledge test score.

### Hindlimb clasping

Hindlimb clasping is a marker of disease progression in a number of mouse models of neurodegeneration, including certain cerebellar ataxias [1].

1. Grasp the tail near its base and lift the mouse clear of all surrounding objects.
2. Observe the hindlimb position for 10 seconds. If the hindlimbs are consistently splayed outward, away from the abdomen, it is assigned a score of 0. If one hindlimb is retracted toward the abdomen for more than 50% of the time suspended, it receives a score of 1. If both hindlimbs are partially retracted toward the abdomen for more than 50% of the time suspended, it receives a score of 2. If its hindlimbs are entirely retracted and touching the abdomen for more than 50% of the time suspended, it receives a score of 3.
3. Place the mouse back into its cage and record its hindlimb clasping score.

### Gait

Gait is a measure of coordination and muscle function.

1. Remove the mouse from its cage and place it on a flat surface with its head facing away from the investigator.
2. Observe the mouse from behind as it walks. If the mouse moves normally, with its body weight supported on all limbs, with its abdomen not touching the ground, and with both hindlimbs participating evenly, it receives a score of 0. If it shows a tremor or appears to limp while walking, it receives a score of 1. If it shows a severe tremor, severe limp, lowered pelvis, or the feet point away from the body during locomotion ("duck feet"), it receives a score of 2. If the mouse has difficulty moving forward and drags its abdomen along the ground, it receives a score of 3.
3. Place the mouse back into its cage and record its gait score.

### Kyphosis

Kyphosis is a characteristic dorsal curvature of the spine that is a common manifestation of neurodegenerative disease in mouse models [2]. It is caused by a loss of muscle tone in the spinal muscles secondary to neurodegeneration.

1. Remove the mouse from its cage and place it on a flat surface.
2. Observe it as it walks. If the mouse is able to easily straighten its spine as it walks, and does not have persistent kyphosis, it receives a score of 0. If the mouse exhibits mild kyphosis but is able to straighten its spine, it receives a score of 1. If it is unable to straighten its spine completely and maintains persistent but mild kyphosis, it receives a score of 2. If the mouse maintains pronounced kyphosis as it walks or while it sits, it is assigned a score of 3.
3. Place the mouse back into its cage and record its kyphosis score.

### Representative results

With a sufficient number of animals, this procedure is capable of detecting phenotype differences between strains and within the same strain over time. For data analysis, calculate the score for each measure by taking the mean of the three measurements in each individual. Measures can be analyzed separately or combined into a composite phenotype score for each individual.

Chart the data and apply the appropriate statistical methods to determine significance. Figure 1 shows results from a composite phenotype assessment of a murine transgenic SCA7 model. In this floxed-SCA7-92Q transgenic model, the human ataxin-7 gene with 92 CAG repeats is flanked by loxP sites and expressed from a bacterial artificial chromosome. The composite phenotype score includes the clasping, ledge walking, gait and kyphosis assessments for a maximum possible score of 12. The progressive SCA7 phenotype in the floxed-SCA7-92Q transgenic mice is demonstrated by an increasing composite phenotype score, which is consistent with the progressive nature of the human disease.


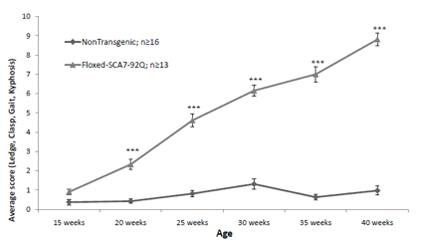
 **Figure 1.** Floxed-SCA7-92Q mice exhibit a progressive SCA7 phenotype that is significantly different from Non-Transgenic littermates beginning at 20 weeks (2-way ANOVA: Bonferroni *post-hoc*; ***P<0.001). Mice were assessed on a 0-3 scale each for ledge test, clasping, gait, and kyphosis. Average composite score for each genotype at each age was calculated. Bars represent SEM.

## Discussion

This protocol is designed to be a sensitive and easily performed evaluation of disease severity in mouse models of cerebellar ataxia. Individual components of the scoring system will be more or less effective in different mouse models of neurodegeneration.

Elements of this scoring system have been effectively used to assess a variety of mouse models of human neurodegenerative disease, including cerebellar ataxias, Huntington s disease and spinobulbar muscular atrophy [1-3]. The ideal combination of tests will depend upon the disorder in question. This protocol was originally designed by the authors to evaluate transgenic mouse models of spinocerebellar ataxia type 7 (SCA7).

In the model of SAC7 employed to generate the data presented here, animals were sacrificed at the age of 40-43 weeks or sooner if the behavioral abnormalities progressed to a stage that an animal is no longer sufficiently mobile to independently maintain nutrition or hydration.
